# Atomic structure and oxygen deficiency of the ultrathin aluminium oxide barrier in Al/AlO_x_/Al Josephson junctions

**DOI:** 10.1038/srep29679

**Published:** 2016-07-12

**Authors:** Lunjie Zeng, Dung Trung Tran, Cheuk-Wai Tai, Gunnar Svensson, Eva Olsson

**Affiliations:** 1 Department of Applied Physics, Chalmers University of Technology, 41296, Göteborg, Sweden; 2 Department of Materials and Environmental Chemistry, Stockholm University, 10691, Stockholm, Sweden

## Abstract

Al/AlO_x_/Al Josephson junctions are the building blocks of a wide range of superconducting quantum devices that are key elements for quantum computers, extremely sensitive magnetometers and radiation detectors. The properties of the junctions and the superconducting quantum devices are determined by the atomic structure of the tunnel barrier. The nanoscale dimension and disordered nature of the barrier oxide have been challenges for the direct experimental investigation of the atomic structure of the tunnel barrier. Here we show that the miniaturized dimension of the barrier and the interfacial interaction between crystalline Al and amorphous AlO_x_ give rise to oxygen deficiency at the metal/oxide interfaces. In the interior of the barrier, the oxide resembles the atomic structure of bulk aluminium oxide. Atomic defects such as oxygen vacancies at the interfaces can be the origin of the two-level systems and contribute to decoherence and noise in superconducting quantum circuits.

Al/AlO_x_/Al Josephson junctions are the building blocks of a wide range of superconducting quantum devices that are key elements for quantum computers, extremely sensitive magnetometers and radiation detectors^1^,^2^,^3^. A fundamental limiting factor for the implementation of Josephson junction based superconducting quantum electronics is the coupling of the coherent quantum state with two-level systems (TLS) accommodated in the amorphous oxide material in the circuits, resulting in noise and decoherence^4^,^5^,^6^,^7^,^8^,^9^,^10^. The TLS in the insulating oxide barrier in Josephson junctions have been considered as a major source of energy dissipation and decoherence in superconducting electric circuits, especially in qubits^11^,^12^,^13^. The most commonly adopted tunnel barrier to date is an ultrathin aluminium oxide AlO_x_ layer formed directly on the Al electrode in Josephson junctions. Many phenomenological theories have been proposed to describe the microscopic nature of the TLS in AlO_x_ tunnel barrier in Josephson junctions and try to correlate the noise and decoherence measurements in qubits with the atomic structure models of the junction^4^,^13^,^14^,^15^,^16^.

Moreover, it has been shown that subgap leakage of the Josephson junctions can come from the interface states due to structural defects at the metal/oxide interfaces in the tunnel junctions^17^.

Despite these advancements in understanding the effect of the structure of barrier oxide on the transport properties of superconducting quantum devices, a direct investigation of the structure of the tunnel barrier, remains challenging due to the nanoscale dimension of the barrier layer and often disordered structure of the barrier oxide. Studies on the stoichiometry and chemical state of thin aluminium oxide films grown on aluminium have been carried out previously^18^,^19^. Recently, an attempt was made to construct an *ab initio* model of a nanosized AlO_x_ barrier in an Al/AlO_x_/Al Josephson junction^20^. Nevertheless, a direct experimental study of the atomic structure of thin AlO_x_ tunnel barriers in a working junction is still missing.

In this study, we directly investigated the microscopic structure of the tunnel barrier in Al/AlO_x_/Al Josephson junctions by using scanning transmission electron microscopy (STEM). Atomic resolution imaging and electron energy loss spectroscopy (EELS) were used to reveal the detailed structure and the chemical bonding of the materials in the junction. By combining nano-beam electron diffraction (NBED), pair distribution function (PDF)^21^ analysis together with reverse Monte Carlo (RMC)^22^ refinement we unravelled the atomic structure of the nanosized AlO_x_ barrier. The technique of combining NBED-PDF and RMC enabled us to treat crystalline Al and amorphous AlO_x_ as integrated parts in the system and take the interfacial interaction between Al and AlO_x_ into account while retrieving the atomic structure information.

The Al/AlO_x_/Al Josephson junction used in this study is fabricated on SiO_2_/Si substrate (Fig. 1a). The mean tunnel barrier thickness in the junction is around 1.8 nm with a standard deviation of the thickness distribution less than 0.5 nm, as presented in a previous study^23^. The areas between the crystalline Al electrodes do not show long-range ordered features (e.g. Fig. 1b), indicating a disordered structure in the barrier layer. The amorphous structure of the barrier oxide was confirmed and investigated by EELS and electron diffraction, which will be presented in the following sections.

To reveal the microscopic structure and chemical bonding in the ultrathin tunnel barrier, spatially-resolved STEM-EELS, in particular, energy loss near edge structure (ELNES) analysis on Al-L_23_ edge was carried out across the tunnel junction, from the top Al electrode into the tunnel barrier and the bottom Al electrode (Fig. 1c).

The spectrum from the metallic Al region was subtracted from the spectrum acquired from the centre of the oxide barrier, which shows most prominent ELNES features from aluminium oxide, using the spatial difference technique^24^. The resulting spectrum (inset of Fig. 1c) shows an edge onset at ~75 eV and 2 main peaks at ~79.6 eV and ~100 eV. There is also a pre-peak feature at around 77.7 eV on the left side of the main peak at ~79.6 eV (inset of Fig. 1(c)). There are many polymorphs of aluminium oxide with similar chemical composition but different atomic structures. The EEL spectrum from barrier oxide is consistent with that obtained from amorphous phase of Al_2_O_3_ and different from crystalline phases^25^. However, the structure of the amorphous phase of aluminium oxide can vary significantly if the fabrication method or the size of the material is different^26^,^27^. In systems containing Al-O bonds, Al-L_23_ edge fine structure is sensitive to the Al coordination, by which different aluminium oxide structures are usually characterized^28^. Amorphous aluminium oxides with different Al coordination have already been reported^26^,^29^,^30^. According to previous studies on the structure of materials containing Al-O bonds, the peak at ~77.6 eV in Al L_23_ ELNES can serve as the fingerprint for tetrahedrally coordinated aluminium in the material^25^,^31^,^32^,^33^. Our EELS data thus suggests that Al atoms in the interior of the ultrathin aluminium oxide barrier in Al/AlO_x_/Al junctions are inclined toward tetrahedral ([AlO_4_]^−5^) coordination rather than octahedral ([AlO_6_]^−9^) or pentahedral ([AlO_5_]^−7^) coordination. The basic structure units of Al and O can be connected via edge- or corner-sharing, forming the oxide without long-range order. Our EELS result is in line with the previous diffraction analysis and Molecular Dynamic (MD) simulations^29^,^34^. However, it has often been reported that a considerable amount of octahedrally and pentahedrally coordinated Al sites is also present in the oxide^26^,^30^,^34^. Furthermore, most of the previous studies were performed on bulk materials (or thick films) and with little contribution from the interfaces considered.

Here we used NBED to further unveil the atomic structure of aluminium oxide in the tunnel barrier (Fig. 2a). PDF analysis (see Methods) was performed on NBED patterns to obtain a distribution of interatomic distances between atoms of both the AlO_x_ barrier (in red, Fig. 2b) and crystalline Al (in blue, Fig. 2b). Most of the peaks in the short range can be reasonably identified: P1: 1.76 Å, Al-O first shell distance; P2: 2.27 Å, Al-O first shell extended distance possibly due to Al-AlO_x_ interaction at the metal/oxide interfaces; P3: 2.83 Å, superimposition of O-O and first shell distance 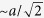 of fcc Al-Al (*a* is the lattice constant of fcc Al, ~4.05 Å); P4: 3.41 Å, Al-Al distances of the barrier oxide and at the interface; P5: 4.00 Å, fcc Al-Al second shell distance ~*a*; P6: 4.95 Å, fcc Al-Al third shell distance ~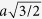. A small peak observed between P5 and P6 can be considered (like P2) as a termination ripple and/or interatomic distance due to Al-AlO_x_ interaction at the interfaces.

In order to extract more detailed structural information, a model was built and compared with the experimental NBED-PDF. The model structure of AlO_x_ was obtained from MC calculation (see Methods) and sandwiched between fcc Al layers to form an initial model of an Al-AlO_x_-Al junction (Fig. 3a). All the featured peaks (P1-P6) in the experimental NBED-PDF can be identified by the model PDF to a first approximation, confirming the above qualitative interpretation of the NBED-PDF (Fig. 3b). According to the atomic resolution STEM images (e.g. Fig. 1b), the Al lattice plane at the Al/AlO_x_ interface is usually highly distorted, indicating strong Al-AlO_x_ interaction at the metal/oxide interfaces. RMC refinement was then carried out by making the model PDF compatible with the experimental NBED-PDF^22^, through adaption of the structure of AlO_x_ and introduction of local dislocations to the metallic Al layers close to the interfaces (see Methods, Fig. 3c,d).

The amorphous structure of the AlO_x_ barrier can be investigated separately in details by removing the crystalline Al layers on both sides of the barrier from the RMC-refined structure of Al-AlO_x_-Al junction. From this separated RMC-refined AlO_x_ model, partial PDFs for Al-O, Al-Al, and O-O pair distances were extracted (Fig. 4a–c). The Al-O distribution after RMC refinement is closer to that of MD bulk liquid Al_2_O_3_, confirming the short-range order established by the Al-O ionic bond (Fig. 4a). The Al-O bond length found by NBED-RMC peaks at 1.77 Å, which is insignificantly different from the value (1.80 Å) of the bulk amorphous aluminium oxide determined experimentally^29^ and by simulations^34^. However, the NBED-RMC Al-O bond length distribution exhibits some shoulders (between 2–4 Å), which have not been reported in either experimental or modelling analyses of the bulk (except the subtle shoulder near 3.5 Å). These features can be the result of the possible Al-AlO_x_ interaction at the interfaces that may be very significant over the small thickness (~1.5–2 nm) of the AlO_x_ barrier. Although the Al-AlO_x_ interaction can not have a dramatic influence on the strong ionic Al-O bond, it may be among the main reasons for the shift of the Al-Al and O-O pair-distance peaks (3.31 Å and 2.81 Å, respectively) (Fig. 4b,c, Table 1). Despite those subtle differences, the bond length distributions in the barrier oxide, especially the Al-O, Al-Al and O-O pair-distances, are well in line with the previous experimental data on bulk amorphous oxide.

Further insights into the nature of the connectivity of the elementary structure units beyond nearest neighbor can be gained via ring structure distribution analysis. The ring structure distribution (Table 1) found in the NBED-RMC of AlO_x_ is very comparable with the results from the MD bulk Al_2_O_3_ where 3-fold, 4-fold and 5-fold rings predominate (a *n*-fold ring has been defined elsewhere^34^). Although 4-fold rings are still present (26.1%) as the largest part in the AlO_x_ structure, they are less compared with the cases of MD liquid (31.6%) and MD amorphous (42.9%). Two-fold rings are found in the NBED-RMC of AlO_x_ with the level of 13.1%, quite similar as the cases of MD liquid (13%) and MD amorphous (9.1%). It is noted that, the presence of 2-fold rings relates to the formation of edge/facet sharing [O_4_]^−8^ tetrahedra. Interestingly, although the initial MC AlO_x_ model was constructed with only corner-sharing tetrahedra, the NBED-RMC refined model has been found to have 78.9% corner-sharing and 21.1% edge-sharing tetrahedra. In the total number of links found between tetrahedra, there are 92.6% corner-links and 7.4% edge-links.

The main difference between the atomic structure of ultrathin barrier oxide and the bulk amorphous aluminium oxide lies in the difference in Al-O coordination. The cut-off distances for structural statistics of the NBED-RMC AlO_x_ were set for Al-O, Al-Al and O-O as 2.5 Å, 4 Å and 3.5 Å, respectively. The AlO_x_ has an average Al-O coordination number of ~3.4 (Fig. 5a,b, Supplementary Fig. 3 and Table 1) while the MD simulations on bulk Al_2_O_3_ gave the values of 4.1 and 4.25 for the liquid and amorphous phases respectively. The low ^[5]^Al fraction (9.4%) in the ultrathin amorphous AlO_x_ tunnel barrier is consistent with the observation that the fraction of Al sites with coordination number 5 decreases with the film thickness in amorphous aluminium oxide thin films^26^. There are almost no octahedrally coordinated Al atoms in the barrier oxide (Fig. 5b and Table 1). In contrast, there are quite many Al atoms in the nanosized AlO_x_ being coordinated with less than 4 O atoms (39.2% Al atoms coordinated with 3 O atoms, 15.6% Al atoms coordinated with 1-2 O atoms). There are only ~35% Al atoms having fully 4 O coordination while in the MD Al_2_O_3_ bulk models it is 66% (liquid) and 76% (amorphous) (Table 1).

The atomic structure of the ultrathin aluminium oxide tunnel barrier in Al/AlO_x_/Al Josephson junctions to a large extent resembles that of the bulk amorphous aluminium oxides in terms of nearest neighbour distance (Fig. 4a–c), bond angle distribution (Supplementary Fig. 2) and network topology of the structure units (Table 1). However, due to the miniaturized dimension of the barrier, the structure of the ultrathin barrier is largely affected by the interfaces. In the interior of the barrier oxide, Al atoms tend to be predominantly tetrahedrally coordinated as evidenced by ELNES analysis and the RMC refined structure of nanosized barrier oxide (Fig. 4e), similar to bulk amorphous aluminium oxide systems. Consequently, the observed abnormally low average Al coordination of the barrier oxide is directly related to the shortage of coordinated O atoms at the surfaces/interfaces of the thin barrier (see visualized structure in Fig. 5a). It is worth noting that it has also been shown before by Auger spectroscopy that a sub-oxide layer often exists at the Al/AlO_x_ interface region^35^. Oxygen deficiency is thus likely present at the interfaces and results in under-coordinated Al sites at the interface region. The oxygen shortage can be related to not only the existence of oxygen vacancies but also the possible relocalization of Al and O atoms in the barrier oxide, in particular, at the interfaces. This relocalization may be evidenced as the emerged shoulders in the Al-O and O-O partial PDFs (Fig. 4a,c) as well as the broadening and shift of the O-O coordination histogram (Supplementary Fig. 3). These structural features can affect the charge carrier tunnelling in many ways. Atomic structure defects in the tunnel barrier are believed to be a major source of TLS in Josephson junction based devices^13^. Additional electronic states can also be formed at the interfaces due to the vacancies, acting as energy dissipation traps and giving rise to subgap leakage of the Josephson junctions^17^.

In summary, the atomic structure of ultrathin AlO_x_ tunnel barrier in Al/AlO_x_/Al junction has been unveiled. In the interior of the barrier, the Al atoms tend to be tetrahedrally coordinated as in bulk oxide, while at the interfaces, Al atoms are largely under-coordinated. The net result is an abnormally low average Al coordination (coordination number ~3.4) of the barrier layer. Otherwise, the aluminium oxide in the tunnel barrier shows similar atomic structure characteristics as bulk amorphous aluminium oxide. Oxygen deficiency at the Al/AlO_x_ interfaces can strongly influence the properties of superconducting devices based on Al/AlO_x_/Al Josephson junctions. We also anticipate the technique we used by combining NBED with RMC simulation can be applied to other systems containing crystal/amorphous interfaces of which the structure information at nanometer scale otherwise is not accessible.

## Methods

### Josephson junction fabrication

The junctions used in this study were grown on Si/SiO_2_ substrates in high vacuum by thermal evaporation. The base pressure of the evaporation system was less than 5×10^−7^ mbar. The SiO_2_ layer was 400 nm thick. A bottom Al film of nominal thickness of 15 nm was deposited with a deposition rate ranging from 9 to 12 Å/s. The sample stage was not cooled or heated up intentionally during the evaporation and oxidation of the Al film. The Al film was thereafter exposed to high-purity (99.99%) O_2_ with fixed pressure (1 mbar) and time (3 min). Subsequently the top Al layer with a nominal thickness of 60 nm was deposited with the same deposition rate as that for the bottom Al film. An Al/AlO_x_/Al Josephson tunnel junction was thus formed. The tunnelling characteristics of the junctions used in this study are representative of large numbers of the junctions analysed in another study^36^. Unpatterned samples (large area trilayer junctions) were used in this study. The area size of the trilayer junctions is ~7 × 7 mm^2^. The detailed structure of the junctions is described in a previous study^23^.

### Transmission electron microscopy

Cross-section TEM specimens were prepared by grinding and polishing the specimen down to ~20 μm, followed by Ar ion milling. The specimens were kept at about −80 °C during milling to minimize the damage from the ion beam. FEI Titan 80–300 equipped with monochromator, probe Cs corrector and Gatan Image filter (GIF) Tridium was used for TEM investigation. Annular dark field (ADF) STEM images were acquired with a 17.5 mrad beam convergence angle and 54–270 mrad detector collection angle. The spatial resolution of the microscope in ADF STEM mode is determined to be ~1 Å. STEM EELS analysis was performed at 300 kV. The collection angle for EELS is ~25 mrad. The TEM sample thickness was measured to be ~35 nm by EELS analysis. Nano beam electron diffraction (NBED) was performed in scanning nanodiffraction mode. Beam convergent angle was adjusted to ~0.5 mrad and beam diameter in this case was around 1.7 nm. To get a NBED pattern from the ultra thin barrier, a STEM image was first acquired. The electron beam was then placed on to the position of the barrier layer in the STEM image. Diffraction patterns with maximum diffraction angle corresponding to Q_max_ ~13 Å^−1^ were used for PDF analysis.

### PDF analysis

PDF analysis was used to interpret the structural information contained in the NBED signals with contributions from the Al electrode layers and aluminium oxide tunnel barrier in the tunnel junction. To handle the possible inhomogeneity and anisotropy of the nano-beam illuminated region, an average scattering data profile was obtained by integration of 4 NBED patterns corresponding to different regions. The scattering data were background-subtracted, scaled and corrected using the electron scattering form factors of Al and O. The normalized PDF was then obtained as the Fourier transform of the scattering data. PDF analysis was carried out using the software SUePDF^37^.

### Monte Carlo modelling

Monte Carlo calculation was used to build initial structure model of the ultrathin amorphous aluminium oxide. A box of AlO_x_ (17 ×_ _27 × 27 Å^3^) in which the number of atoms inside the box was restricted by a density of 3.12 g/cm^3^ was built. A Delaunay network of O was first generated with a minimum allowed O-O distance of 2.6 Å. Al atoms were then put inside the selected [O_4_]^−8^ tetrahedra, which do not share more than one O vertex atom with each other. Al and O positions were refined until their minimum interatomic distances reached the predefined criteria: Al–Al: 3.1 Å, Al-O: 1.55 Å, and O-O: 2.6 Å. The MC-AlO_x_ model ended up with 456 Al and 681O atoms.

### Reverse Monte Carlo refinement

**Figure 1:**
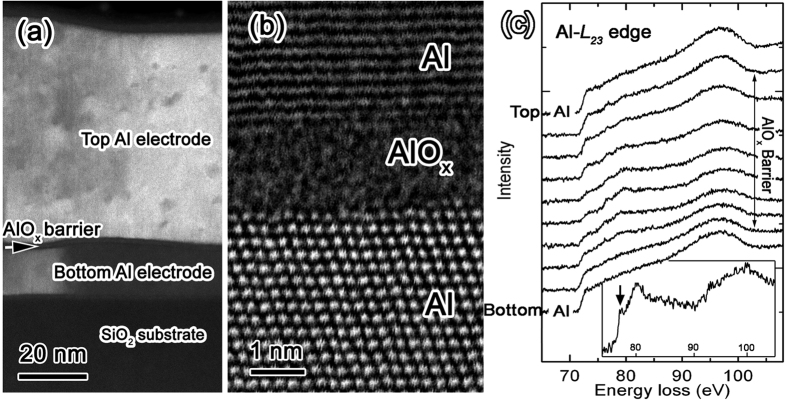
Structure of an Al/AlO_x_/Al junction from scanning transmission electron microscopy (STEM) imaging and electron energy loss spectroscopy (EELS) analysis. (**a**) A cross-sectional ADF STEM image showing different layers in a typical Al/AlO_x_/Al Josephson junction with AlO_x_ formed by thermal oxidation directly on the bottom Al electrode. (**b**) A high resolution ADF STEM image of a junction area showing the tunnel barrier. Lattice fringes and atomic columns from crystalline Al region are visible. (**c**) Al-L_23_ EELS line-profile obtained across the tunnel junction, from top Al electrode, aluminium oxide tunnel barrier to bottom Al electrode. The inset shows the Al-L_23_ ELNES signal from the centre of the barrier after subtracting the contribution from the Al electrode.

**Figure 3:**
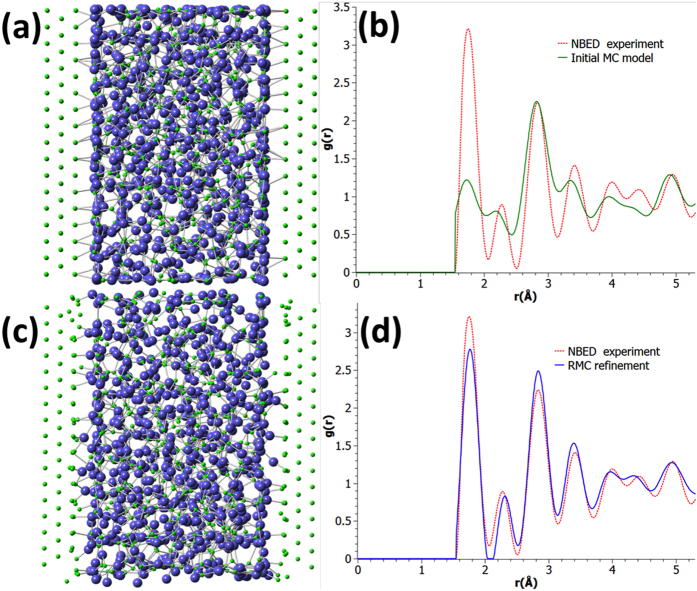
Structure models of Al-AlO_x_-Al junction and the corresponding PDFs. (**a**) Initial structure model built by Monte Carlo (MC) simulation. (**b**) Initial MC model’s PDF compared with the experimental NBED-PDF. (**c**) RMC-refined model. (**d**) RMC-refined model’s PDF fitted to the experimental NBED-PDF.

Structure models of Al/AlO_x_/Al Josephson junction were built and compared with the experimental NBED-PDF. The model structure of AlO_x_ was obtained from MC calculation and sandwiched with *n* (*n* = 2, 3, 4, 5, and 6) layers of {220} fcc Al to form an initial model of an Al^*n*^-AlO_x_-Al^*n*^ junction. According to the atomic resolution STEM images (e.g. Fig. 1b), the Al lattice plane at the Al/AlO_x_ interface is usually highly distorted, indicating strong Al-AlO_x_ interaction at the metal/oxide interfaces. Reverse Monte-Carlo (RMC)^22^ refinement was then carried out for all the Al^*n*^-AlO_x_-Al^*n*^ junction models (Fig. S1) against the experimental NBED-PDF. The refinements were performed by moving the atoms in the AlO_x_ barrier and those in the Al layer at the Al-AlO_x_ interface. The minimum interatomic distance constraints were: Al^(AlO^_x_)-Al^(AlO^_x_): 3.1 Å, Al^(AlO^_x_)-Al^(Al)^: 2.7 Å, O-O: 2.6 Å, Al-O: 1.55 Å. The RMC refinement has made the model PDF compatible with the experimental NBED-PDF (Fig. 3(d)) through adaption of the structure of AlO_x_ and introduction of local dislocations to the metallic Al layers close to the interfaces. Based on the examination of the fitting of the PDF and the final structure model after the RMC refinements (Figs S1 and S2), the RMC-refined Al^3^-AlO_x_-Al^3^ junction model with three layers of fcc Al was chosen as the best representative. It can be considered that the match between the RMC-refined model and the experiment is limited by experimental errors, which mainly include multiple scattering from the crystalline Al and beam convergence^38^.

## Additional Information

**How to cite this article**: Zeng, L. J. *et al.* Atomic structure and oxygen deficiency of the ultrathin aluminium oxide barrier in Al/AlO_x_/Al Josephson junctions. *Sci. Rep.* **6**, 29679; doi: 10.1038/srep29679 (2016).

## Supplementary Material

Supplementary Information

## Figures and Tables

**Figure 2:**
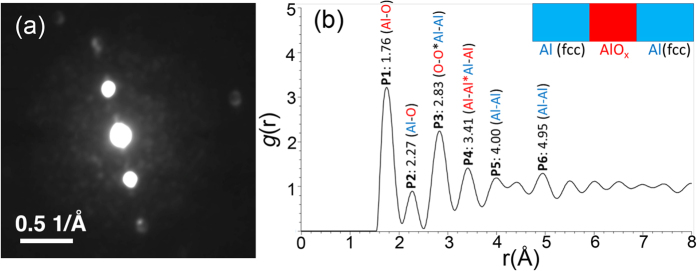
Nanobeam electron diffraction (NBED) and pair distribution function (PDF) analysis of the barrier oxide. (**a**) A typical NBED pattern of the AlO_x_ barrier. It includes contributions from the adjacent crystalline Al as evidenced by the Bragg spots from Al. Diffused and speckle intensities are from the amorphous barrier oxide. (**b**) pair distribution function obtained from a set of NBED data. Peaks P1-P6 are identified as the attributes of interatomic pair distances, involving both the AlO_x_ barrier (in red) and crystalline Al layers (in blue).

**Figure 4:**
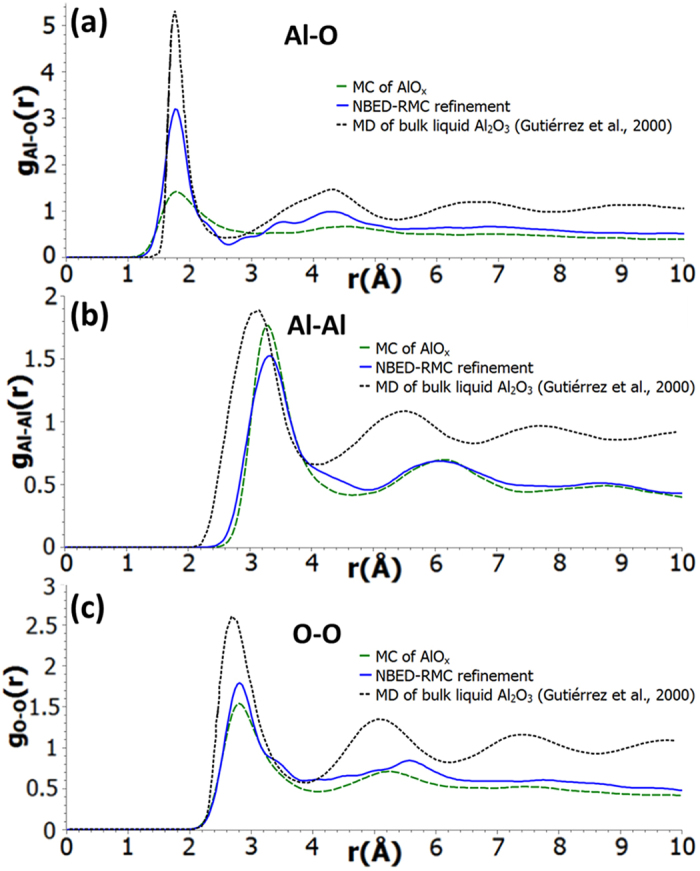
Atomic structure of the barrier oxide in the Al/AlO_x_/Al junction. (**a**–**c**) Partial PDFs of NBED-RMC refined AlO_x_ (blue-solid) compared with partial PDFs of initial AlO_x_ structure model (green-dash) and previous MD simulated bulk liquid Al_2_O_3_ (black-dash).

**Figure 5:**
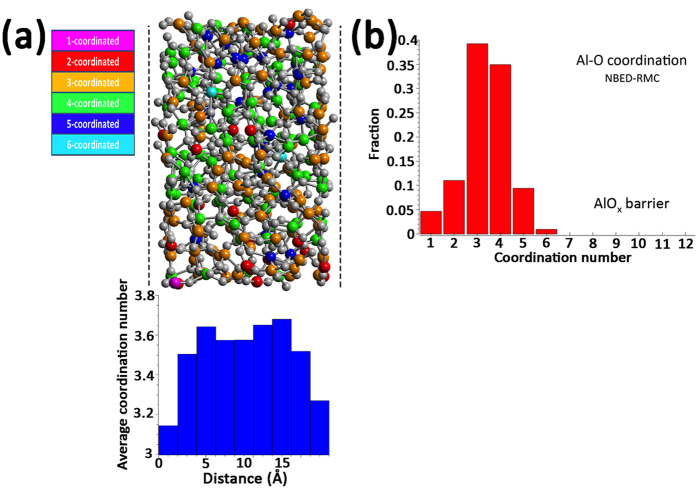
Al-O coordination in the aluminium oxide barrier in the Al/AlO_x_/Al junction. (**a**) the refined atomic structure model of aluminium oxide tunnel barrier with the visualization of Al (coloured)–O (grey) coordination. Al atoms with different colours have different O coordination numbers. Dashed lines indicate the positions of the Al/AlO_x_ interfaces. The profile of 2 Å-column averaged coordination number of Al atoms across the AlO_x_ barrier is shown below the atomic structure model. The coordination number is averaged along the direction parallel to the Al/AlO_x_ interfaces and with a 2 Å step along the direction perpendicular to the interfaces. (**b**) Al-O coordination number distribution in the barrier oxide.

**Table 1:** Structural analysis of NBED-RMC nano-AlO_x_ in comparison with previous structure data on liquid bulk Al_2_O_3_ and amorphous bulk Al_2_O_3._

Model	Pair distance peak (Å)			Al-O coordination (%)				Ring distribution (%)				
	Al-O	Al-Al	O-O	3	4	5	6	2	3	4	5	6
NBED-RMC nano-AlO_x_	1.77	3.31	2.81	39.2	34.9	9.4	0.9	13.1	22	26.1	23.2	4.2
MD Liquid bulk Al_2_O_3_^*^	1.75	3.15	2.75	13	66	20	≤1	13	24.6	31.6	22.6	7.5
MD Amorphous bulk Al_2_O_3_^**^	1.76	3.12	2.75	0	76	22	2	9.1	33.7	42.9	13.2	1
XRD amorphous bulk Al_2_O_3_^***^	1.80	3.20	2.80	20	56	22	0					

*Gutiérrez, G. *et al.* *Phys. Rev. E*, 61, 2723 (2000).

**Gutiérrez, G. *et al.* *Phys. Rev. B*, 65, 104202 (2002).

***Lamparter, P. and Kniep, R., *Physica B* 234–236, 405 (1997).
