# Supplementary material for: Atomic structure and oxygen deficiency of the ultrathin aluminium oxide barrier in Al/AlOx/Al Josephson junctions
Source: Sci Rep. 2016 Jul 12;6:29679. doi: 10.1038/srep29679 (PMC4940733; doi:10.1038/srep29679)
Supplement: Supplementary Information [file srep29679-s1.doc]

**Supplementary text**

**Atomic structure and oxygen deficiency of the ultrathin aluminum oxide barrier in Al/AlO_x_/Al Josephson junctions**

Lunjie Zeng^1^, Dung Trung Tran^2^, Cheuk-Wai Tai^2^, Gunnar Svensson^2^, Eva Olsson^1^

*^1^Department of Applied Physics, Chalmers University of Technology, 41296, Göteborg, Sweden*

*^2^Department of Materials and Environmental Chemistry, Stockholm University, 10691, Stockholm, Sweden*

Reverse Monte Carlo refinement:

Fig. S1 shows the structure models of Al/AlO_x_/Al Josephson junction built from Monte Carlo (MC) simulation and compared with the experimental nano beam electron diffraction-pair distribution function (NBED-PDF). The model structure of AlO_x_ was sandwiched with $n$ ($n$= 2, 3, 4, 5, and 6) layers of {220} fcc Al to form an initial model of an Al^n^-AlO_x_-Al^n^ junction. Reverse Monte-Carlo (RMC) refinement was then carried out for all the Al^n^-AlO_x_-Al^n^ junction models. The result for the structure model with no crystalline Al (n=0) included is also shown in Fig. S1.

Bond angle distribution:

Bond angle distributions for Al-Al-Al, Al-Al-O, Al-O-Al, Al-O-O, O-Al-O, and O-O-O bonds were calculated based on the structure of the barrier oxide obtained from RMC refinement (Fig. S2). These distributions are also compared with the results from liquid Al_2_O_3_ reported previously ^1^ and also those calculated from the initial structure model before RMC refinement (Fig. S2). The main features in the distributions from the RMC refined structure follow those in liquid Al_2_O_3_ closely. The structure models have relatively small amount of atoms comparing to bulk structure, so the distributions all show high noise level. Comparing to the bulk structure, there are some extra fine features in the bond angle distribution of RMC refined nanosized aluminum oxide. For example, the peaks at around 50° in both Al-Al-O and Al-O-O angle distributions are not visible in bulk structure.

Coordination number distribution:

Fig. S3 shows coordination number distributions for Al-O and O-O coordination obtained from the barrier oxide structure. These distributions are also compared with the results from bulk amorphous aluminum oxide reported previously ^1,2^.


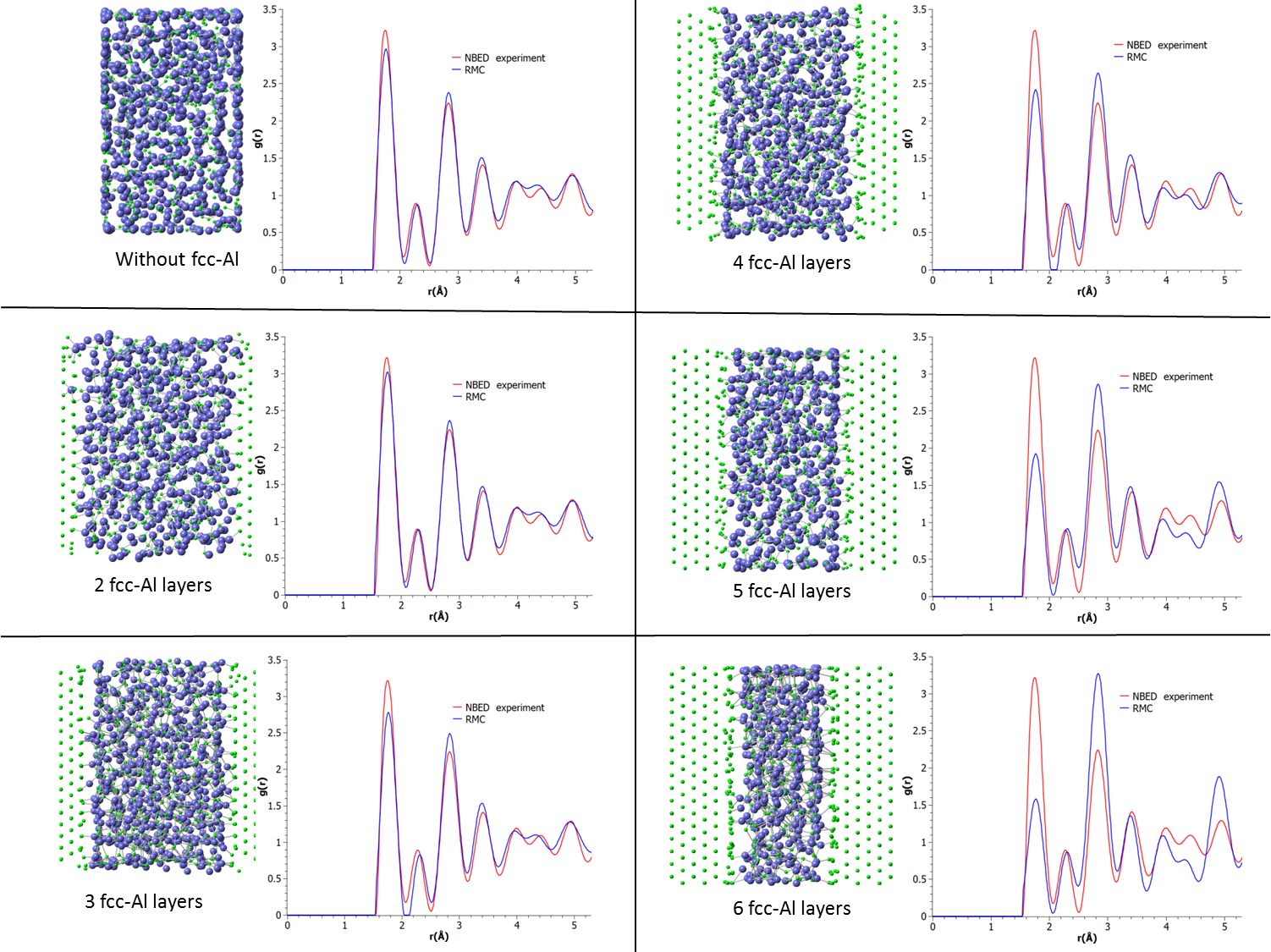


**Figure S1.** Initial MC models with n (n=0 and 2-6) {220} layers of fcc-Al and the corresponding NBED-RMC refinements.


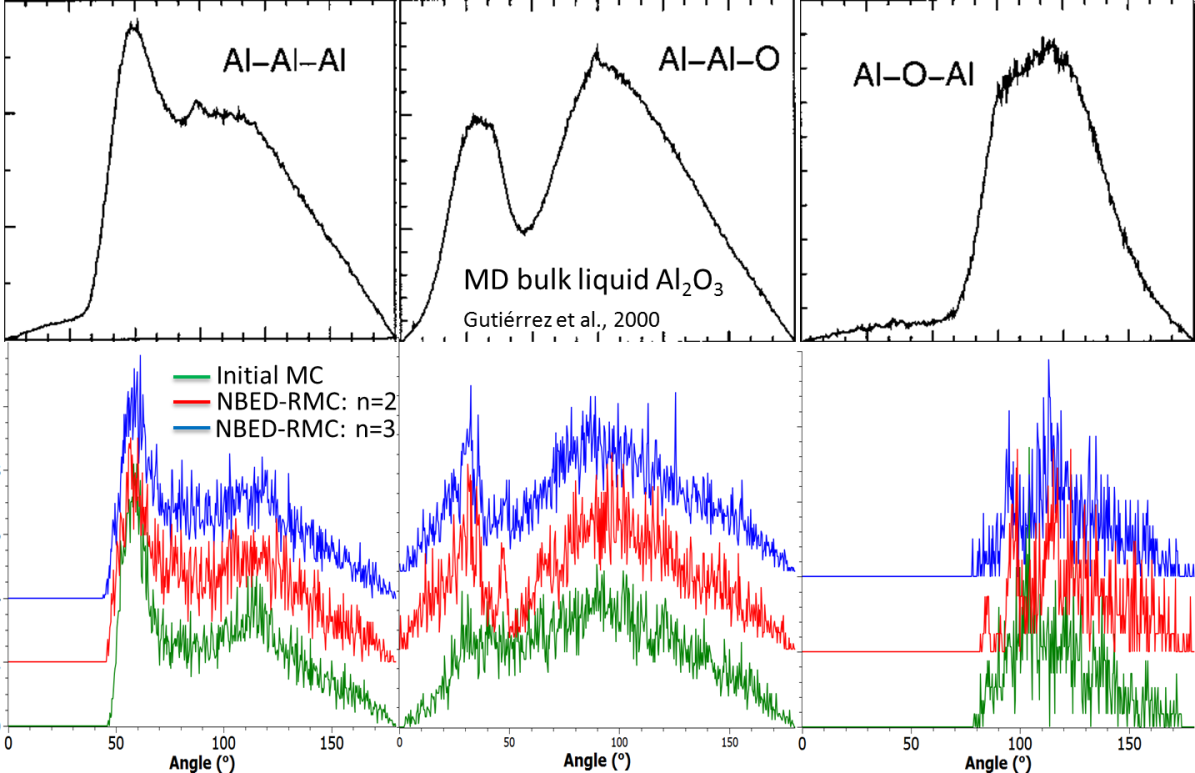


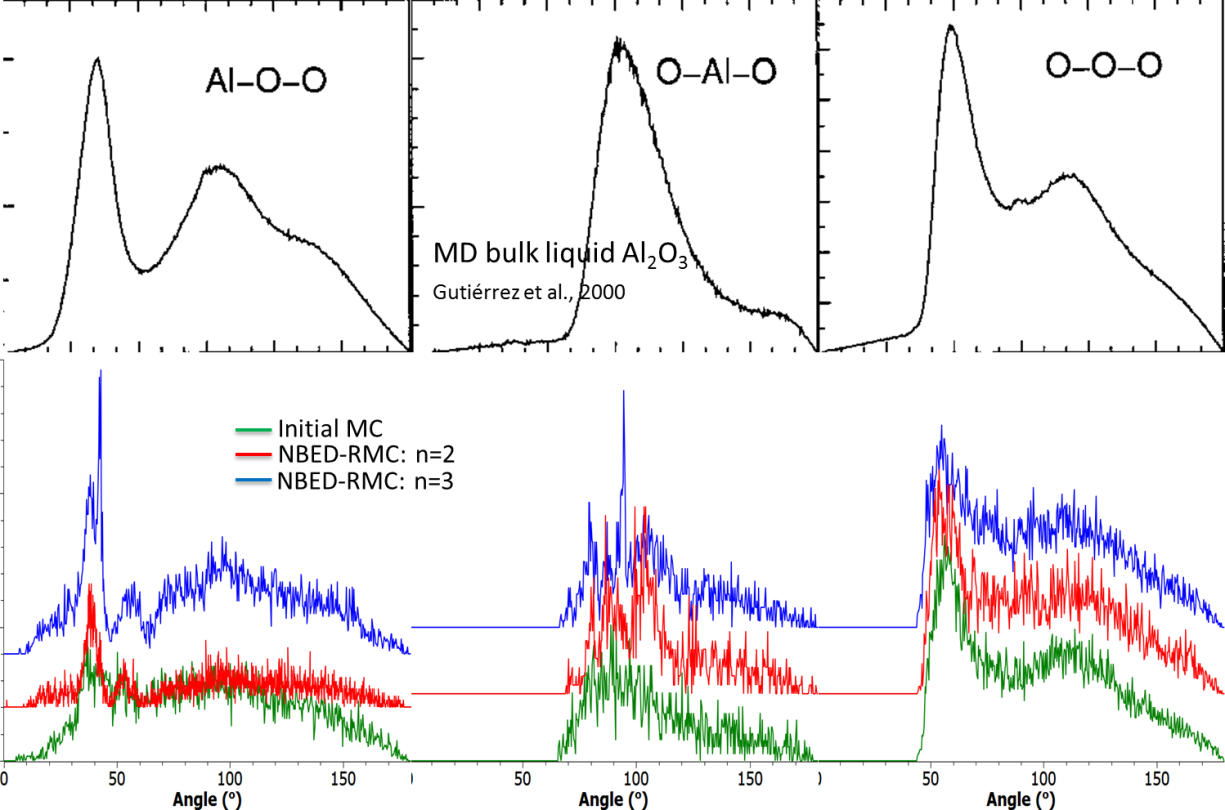


**Figure S2.** Bond angle distributions of AlO_x_ models which have been refined with n = 2 (red) and n = 3 (blue), compared with the initial MC model and the previous MD bulk liquid Al_2_O_3_.


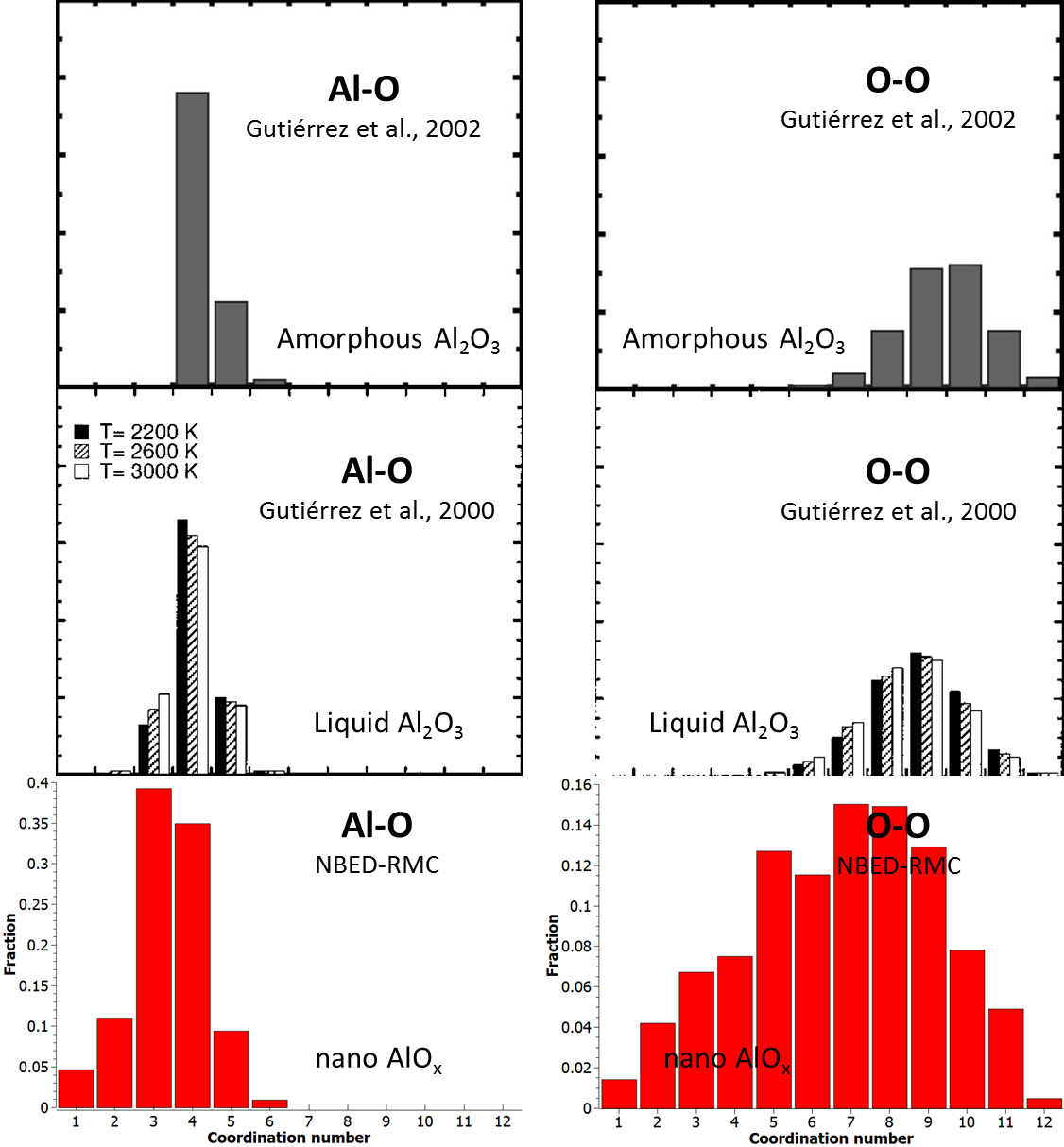


**Figure S3.** Distribution histograms for Al-O and O-O coordination numbers of the NBED-RMC AlO_x_ compared with the cases of MD bulk liquid and amorphous Al_2_O_3_.

References:

1. Gutiérrez, G., Belonoshko, A. B., Ahuja, R. & Johansson, B. Structural properties of liquid Al 2 O 3 : A molecular dynamics study. *Phys. Rev. E* **61,** 2723–2729 (2000).

2. Gutiérrez, G. & Johansson, B. Molecular dynamics study of structural properties of amorphous Al 2 O 3. *Phys. Rev. B* **65,** 104202 (2002).
